# Controlled Exposures to Air Pollutants and Risk of Cardiac Arrhythmia

**DOI:** 10.1289/ehp.1307337

**Published:** 2014-03-25

**Authors:** Jeremy P. Langrish, Simon J. Watts, Amanda J. Hunter, Anoop S.V. Shah, Jenny A. Bosson, Jon Unosson, Stefan Barath, Magnus Lundbäck, Flemming R. Cassee, Ken Donaldson, Thomas Sandström, Anders Blomberg, David E. Newby, Nicholas L. Mills

**Affiliations:** 1 University of Edinburgh, University/BHF Centre for Cardiovascular Science, Edinburgh, United Kingdom; 2 Department of Public Health and Clinical Medicine, Division of Medicine/Respiratory Medicine, Umeå University, Umeå, Sweden; 3 National Institute for Public Health and the Environment (RIVM), Bilthoven, the Netherlands

## Abstract

Background: Epidemiological studies have reported associations between air pollution exposure and increases in cardiovascular morbidity and mortality. Exposure to air pollutants can influence cardiac autonomic tone and reduce heart rate variability, and may increase the risk of cardiac arrhythmias, particularly in susceptible patient groups.

Objectives: We investigated the incidence of cardiac arrhythmias during and after controlled exposure to air pollutants in healthy volunteers and patients with coronary heart disease.

Methods: We analyzed data from 13 double-blind randomized crossover studies including 282 participants (140 healthy volunteers and 142 patients with stable coronary heart disease) from whom continuous electrocardiograms were available. The incidence of cardiac arrhythmias was recorded for each exposure and study population.

Results: There were no increases in any cardiac arrhythmia during or after exposure to dilute diesel exhaust, wood smoke, ozone, concentrated ambient particles, engineered carbon nanoparticles, or high ambient levels of air pollution in either healthy volunteers or patients with coronary heart disease.

Conclusions: Acute controlled exposure to air pollutants did not increase the short-term risk of arrhythmia in participants. Research employing these techniques remains crucial in identifying the important pathophysiological pathways involved in the adverse effects of air pollution, and is vital to inform environmental and public health policy decisions.

Citation: Langrish JP, Watts SJ, Hunter AJ, Shah AS, Bosson JA, Unosson J, Barath S, Lundbäck M, Cassee FR, Donaldson K, Sandström T, Blomberg A, Newby DE, Mills NL. 2014. Controlled exposures to air pollutants and risk of cardiac arrhythmia. Environ Health Perspect 122:747–753; http://dx.doi.org/10.1289/ehp.1307337

## Introduction

Exposure to air pollution is a major public health concern and is associated with morbidity and mortality from cardiorespiratory diseases (Brook et al. 2010). Indeed, on a population level, exposure to combustion-derived particulate air pollution from traffic is recognized as a major trigger for myocardial infarction (Nawrot et al. 2011). With growing concern over the effects of exposure to air pollutants for the general public and susceptible patient populations, there is an increasing interest in defining the risks and underlying vascular and inflammatory mechanisms that may explain these observed associations.

The cardiovascular effects of air pollution are complex and include effects on vascular endothelial function, thrombosis, platelet function, and atherogenesis, as well as changes in blood pressure and cardiac autonomic control (Langrish et al. 2012a). Indeed, changes in autonomic control of the heart, measured by heart rate variability (HRV), have been widely studied in the air pollution literature, and a recent meta-analysis of 18,667 participants enrolled in 29 studies reported an inverse relationship between measures of HRV and exposure to particulate air pollution (Pieters et al. 2012). Reduced HRV represents a withdrawal of cardiac vagal tone or an increase in sympathetic tone and is a predictor of poor prognosis in patients recovering from myocardial infarction and in patients with cardiac failure (Task Force of the European Society of Cardiology and the North American Society of Pacing and Electrophysiology 1996) and may increase the risk of cardiac arrhythmias in these at-risk patients (Odemuyiwa et al. 1991). Recent evidence has linked activity of the autonomic nervous system and atrial electrical properties with the triggering of atrial arrhythmias such as atrial fibrillation (AF) and flutter (Arora 2012; Lo et al. 2011; Park et al. 2012). There is some limited epidemiological evidence linking exposure to air pollutants to both ventricular and supraventricular arrhythmias (Brook et al. 2010), although these associations are not consistent (Gold and Mittleman 2013).

In the present study, we explored our database of continuous electrocardiographic (ECG) recordings made during controlled exposures to a range of air pollutants to determine whether there is evidence of an increase in the short-term risk of arrhythmia during such exposures.

## Methods

Data were extracted from 13 consecutive randomized double-blind crossover studies including healthy volunteers and patients with coronary heart disease from 2004 to 2013 (Table 1). All trials were reviewed and approved by the appropriate local ethics review boards of the NHS Research Ethics Service (Edinburgh, UK), Umeå University (Umeå, Sweden), or the Chinese Academy of Medical Sciences (Beijing, China). All participants gave written informed consent in accordance with the Declaration of Helskini. All healthy volunteers had a normal 12-lead ECG and cardiovascular response to exercise determined at screening. Patients with coronary heart disease were excluded if they had a history of arrhythmia, severe coronary disease without revascularization, significant valvular heart disease or left ventricular systolic dysfunction, conduction abnormality on a resting 12-lead ECG, uncontrolled hypertension, or an acute coronary syndrome within the previous 3 months. Participants were exposed to a variety of air pollutants in controlled-exposure studies or in ambient settings [with the use of a highly efficient facemask to provide a control (Langrish et al. 2009)]. Detailed monitoring of personal exposure was performed and continuous ECGs recorded to allow for the assessment of cardiac arrhythmia (Barath et al. 2010; Cruts et al. 2008; Langrish et al. 2009, 2012b; Mills et al. 2005, 2007, 2008, 2011b).

**Table 1:** Baseline characteristics and medical history of participants included in studies.

Parameter	Controlled exposure					Ambient exposure
	Diesel exhaust	Wood smoke	O_3_^–^	CAPs	Engineered carbon NPs	Personal monitoring
Healthy volunteers
Baseline characteristics
No. of volunteers	80	29	15	17	14	14
Sex (male)	74 (93)	21 (72)	15 (100)	17 (100)	14 (100)	2 (14)
Age (years)	25 (18–24)	26 (20–35)	25 (22–30)	48 (21–69)	21 (20–44)	27 (20–45)
BMI (kg/m^2^)	24 ± 3	25 ± 4	NA	25 ± 3	23 ± 2	21 ± 2
Pulse (bpm)	65 ± 12	62 ± 13	68 ± 13	63 ± 10	74 ± 8	79 ± 3
SBP (mmHg)	138 ± 17	122 ± 14	150 ± 15	139 ± 20	132 ± 12	113 ± 8
DBP (mmHg)	72 ± 9	72 ± 8	74 ± 8	77 ± 8	68 ± 8	73 ± 6
Hemoglobin (g/dL)	147 ± 11	146 ± 9	148 ± 9	144 ± 10	148 ± 11	NA
Creatinine (μmol/L)	76 ± 12	78 ± 20	NA	NA	NA	NA
Patients
Baseline characteristics
No. of patients	37	NA	NA	12	NA	93
Sex (male)	33 (89)	NA	NA	12 (100)	NA	80 (86)
Age (years)	63 (51–80)	NA	NA	59 (45–68)	NA	63 (45–77)
BMI (kg/m^2^)	27 ± 3	NA	NA	28 ± 3	NA	26 ± 3
Pulse (bpm)	57 ± 8	NA	NA	53 ± 5	NA	67 ± 10
SBP (mmHg)	139 ± 20	NA	NA	138 ± 10	NA	131 ± 17
DBP (mmHg)	77 ± 8	NA	NA	80 ± 10	NA	79 ± 10
Hemoglobin (g/dL)	140 ± 11	NA	NA	148 ± 7	NA	142 ± 11
Creatinine (μmol/L)	78 ± 12	NA	NA	101 ± 12	NA	76 ± 16
Medical history
Previous MI	23 (62)	NA	NA	7 (58)	NA	68 (73)
Diabetes mellitus	0 (0)	NA	NA	0 (0)	NA	43 (46)
Hypercholesterolemia	25 (68)	NA	NA	12 (100)	NA	40 (43)
Hypertension	8 (22)	NA	NA	4 (33)	NA	75 (81)
Angina pectoris	14 (38)	NA	NA	5 (42)	NA	65 (70)
Medication use
Aspirin	36 (97)	NA	NA	12 (100)	NA	87 (94)
Clopidogrel	3 (8)	NA	NA	0 (0)	NA	16 (17)
Beta-blocker	26 (70)	NA	NA	11 (92)	NA	67 (72)
Statin	32 (87)	NA	NA	12 (100)	NA	73 (79)
ACE inhibitor	13 (35)	NA	NA	1 (8)	NA	28 (30)
Abbreviations: ACE, angiotensin-converting enzyme; BMI, body mass index; CAPs, concentrated ambient particles; DBP, diastolic blood pressure; MI, myo­cardial infarction; NA, not applicable; NPs, nano­particles; SBP, systolic blood ­pressure. Values are expressed as *n* (%), mean ± SD, or median (IQR), as appropriate.						

In controlled-exposure studies, participants were exposed using a randomized, double-blind controlled crossover design to either filtered air or to the experimental pollutant during intermittent exercise. The exposure time varied across these studies and is documented below. Each study visit was separated from the next by at least 7 days.

*Controlled and ambient exposure generation*. Diesel-exhaust exposures. Controlled exposures to dilute diesel exhaust were performed in purpose-built exposure chambers in Umeå, Sweden (Mills et al. 2005), and in Edinburgh, United Kingdom, through a collaboration with the National Institute for Public Health and the Environment (RIVM), the Netherlands, as described previously (Mills et al. 2011b). In Sweden, diesel exhaust was produced by a diesel engine (model TD45, 4.5 L, 4 cylinders; Volvo, Gothenburg, Sweden) under idling (Mills et al. 2005, 2011b) or city-cycle conditions (Barath et al. 2010). More than 90% of the exhaust was shunted away and the remainder mixed with filtered air and fed into a purpose-built whole-body exposure chamber at steady-state concentration. Air was sampled in the participant’s breathing zone and analyzed continuously for particle mass concentration, particle number concentration, oxides of nitrogen (NO_x_), carbon monoxide (CO), and total hydrocarbons (Mills et al. 2005). In Edinburgh, diesel exhaust was produced from a diesel electricity generator (4 cylinder, 2.2 L, 500 rpm; Deutz, Cologne, Germany) and was diluted as above before being fed into a modified body-box exposure chamber (Mills et al. 2011b). The exposures were standardized with a target particulate matter mass concentration of 300 μg/m^3^. Participants were exposed to the diesel exhaust and filtered air for 1 hr during intermittent exercise on a bicycle to generate an average minute ventilation of 20 L/min/m^2^ body surface area.

Wood-smoke exposures. Wood smoke was generated using a common Nordic wood stove (chimney stove) in a controlled incomplete combustion firing procedure (Unosson et al. 2013). Birch wood logs with a moisture content of 16–18% were inserted every 5–15 min to maintain a high burn rate with repeated air-starved conditions. The wood smoke was diluted with filtered air (HEPA filter and activated carbon filter) in three steps and continuously fed into a controlled environment exposure chamber (15.3 m^3^) to achieve a steady-state concentration. The atmosphere in the chamber was monitored for gaseous pollutants using continuous measurement of NO_x_ and CO. PM_1_ (particulate matter with an aerodynamic diameter of ≤ 1 μm) mass concentration was measured on-line using a tapered element oscillating microbalance (TEOM 1400; Thermo Scientific, Waltham, MA, USA) equipped with a PM_1_ pre-cyclone. Integrated with the TEOM, a filter (Teflon) sampling line was used to determine the particle mass concentration gravimetrically. The exposures were standardized with a target PM_1_ mass concentration of 300 μg/m^3^ for 3 hr (*n* = 14) or 1,000 μg/m^3^ for 1 hr (*n* = 15). As before, participants were exposed to wood smoke during intermittent exercise to generate an average minute ventilation of 20 L/min/m^2^ body surface area.

Ozone (O_3_^–^) exposures. O_3_^–^ was generated using an O_3_^–^ generator (500 MM; Fischer Scientific, Schwerte, Germany) and the O_3_^–^ concentration was measured continuously in the participant’s breathing zone using a photometric O_3_^–^ analyzer (model 1108; Dasibi Environmental Corp., CA, USA). During the exposures, ambient air was continuously drawn through the chamber at a ventilation rate of 30 m^3^/hr (Blomberg et al. 1999). Temperature and relative humidity were maintained at 20°C and 50%, respectively. The exposures were standardized to an O_3_^–^ concentration of 300 ppb for 75 min. Again participants performed intermittent exercise during the exposure to maintain an average minute ventilation of 20 L/min/m^2^ body surface area.

Concentrated ambient particle (CAP) exposures. A versatile aerosol concentration enrichment system (VACES) concentrator within a mobile ambient particle concentrator exposure laboratory sited outside the Royal Infirmary of Edinburgh (Edinburgh, UK), as used to deliver CAP exposures as described previously (Mills et al. 2008). Incoming ambient air (500 L/min) was saturated with water vapor to increase the size of ultrafine and fine particles before being passed through five parallel virtual impactors, each operating at 100 L/min. This increase in size, and therefore mass, ensured the particles had sufficient momentum to pass through the impactors, exiting in the minor flow (5 L/min) in which the particle concentration is enriched by a factor of 10- to 20-fold. The outward minor flow from the five impactors (25 L/min) was desaturated by silica gel dryers to restore the particles to their original size, and diluted with filtered air before delivery into the human exposure chamber (50 L/min). Air was sampled in the participant’s breathing zone and analyzed continuously for temperature, humidity, particle mass concentration, particle number concentration, NO_x_, CO, sulfur dioxide (SO_2_), and O_3_^–^ (Mills et al. 2008). Participants were exposed to CAPs at a target concentration of 200 μg/m^3^ for 2 hr during intermittent exercise to generate an average minute ventilation of 20 L/min/m^2^ body surface area.

Exposure to engineered carbon nanoparticles (NPs). An aerosol of carbon NPs was generated from graphite electrodes using an electric spark discharge generator (model CFG1000; Palas GmbH, Karlsruhe, Germany) in an atmosphere of pure argon. The output of the generator was mixed with filtered air, passed through an impactor with a cutoff of 0.1 μm, and fed into a whole-body exposure chamber (Mills et al. 2011b). Participants were exposed to the carbon particles for 2 hr with a target exposure of 4 × 10^6^ particles/cm^3^ during intermittent exercise to generate an average minute ventilation of 20 L/min/m^2^ body surface area.

Ambient exposures and personal monitoring. On two occasions in two randomized open-label controlled crossover studies, participants were randomized to wear no mask or a highly efficient occupational facemask (Dust Respirator 8812; 3M, St. Paul, MN, USA) as described previously (Langrish et al. 2009, 2012b). The facemask visit was deemed the “control” visit for these analyses. Participants were asked to walk for 2 hr in a city center location in Beijing, China, between 0800 and 1000 hours. Exposure to ambient air pollutants was measured using portable monitoring equipment mounted in a backpack. PM_2.5_ mass (≤ 2.5 μm in aerodynamic diameter) and number concentrations, CO and SO_2_ levels, temperature, and humidity were recorded (Langrish et al. 2009). Physical activity was recorded using a global positioning system monitor within the backpack to ensure exercise performed on each visit was equivalent.

*Continuous ECGs and arrhythmia analysis*. Continuous ECGs were recorded from all participants (model 90217; Spacelabs Healthcare, Hertford, UK) during the exposure and for the subsequent 24 hr, except in one diesel-exhaust study where the recordings were for 3 hr postexposure (*n* = 10) and one wood-smoke study where the recordings were for 8 hr postexposure (*n* = 14). Data were analyzed using the Pathfinder Digital 700 Series Analysis System (Delmar Reynolds, Hertford, UK). Arrhythmias were identified using an automated algorithm and confirmed manually by trained operators.

*Data analysis and statistics*. The number of participants with observed arrhythmia was determined during the pollutant and control exposure periods and compared using the chi-square test, and odds ratios (ORs) were calculated. The numbers of arrhythmias per participant during pollutant and control exposure periods were compared using the Wilcoxon matched pairs signed rank test. All analyses were performed using GraphPad Prism (version 5 for Macintosh; GraphPad Software, San Diego, CA, USA). Statistical significance was taken as a two-sided *p*-value of < 0.05. Data are expressed as median [interquartile range (IQR)] or mean ± SD, as appropriate.

## Results

We identified 282 participants (140 healthy volunteers and 142 patients with coronary heart disease; Table 1) who had been exposed to dilute diesel exhaust (*n* = 117) (Barath et al. 2010; Cruts et al. 2008; Mills et al. 2005, 2007, 2011b), wood smoke (*n* = 29), O_3_^–^ (*n* = 15), CAPs (*n* = 29) (Mills et al. 2008), and engineered carbon NPs (*n* = 14) (Mills et al. 2011b) in controlled-exposure studies and ambient air pollution (*n* = 107) in Beijing, China (Langrish et al. 2009, 2012b) (Table 2). The mean recording time was 22 ± 5 hr, and there were > 12,500 hr of ECG data in total.

**Table 2:** Exposure parameters for air pollution exposures.

Exposure (min)	Group	PM_2.5_ (μg/m^3^)	PM_10_ (μg/m^3^)	Particle count (× 10^4^/cm^3^)	CO (ppm)	NO_x_ (ppm)	NO (ppm)	NO_2_ (ppm)	O_3_^–^ (ppm)	THC (ppm)
Diesel exhaust
60 (*n *= 64)	HV	NA	307	79	6.57	4.08	4.45	1.10	NA	3.0
60 (*n *= 37)	Patients	NA	294	103	3.82	3.29	2.46	0.83	NA	2.8
120 (*n *= 16)	HV	NA	363	120	3.50	0.60	0.40	0.20	NA	NA
Ambient (median)
120 (*n *= 14)	HV	86	NA	2.4	NA	NA	NA	NA	NA	NA
120 (*n *= 93)	Patients	89	NA	4.4	NA	NA	NA	NA	NA	NA
Wood smoke
60 (*n *= 15)	HV	895 (PM_1_)	NA	NA	15.32	NA	0.53	NA	NA	NA
180 (*n *= 14)	HV	314 (PM_1_)	NA	NA	26.00	0.41	NA	NA	NA	NA
O_3_^–^
75 (*n *= 15)	HV	NA	NA	NA	NA	NA	NA	NA	0.30	NA
CAPs
120 (*n *= 29)	HV & patients	NA	190	9.9	0.02	0.01	0.01	0.01	NA	NA
Engineered carbon NPs
120 (*n *= 14)	HV	NA	70	387	NA	NA	NA	NA	NA	NA
Abbreviations: HV, healthy volunteers; NA, not applicable; NO, nitric oxide; NO_2_, nitrogen dioxide; PM_1_, ≤ 1 μm in aerodynamic diameter; PM_10_, ≤ 10 μm in aerodynamic diameter; THC, total hydrocarbons. Values are expressed as mean, except as noted.										

**Figure 1:**
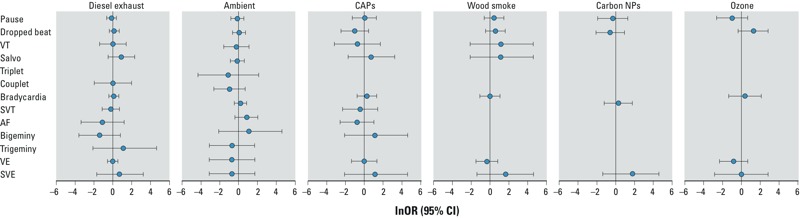
Forest plots showing the risk of arrhythmias during and after exposure to air pollutants compared with a control air exposure (or in the presence of a highly efficient facemask for the ambient exposures). Abbreviations: SVE, supraventricular ectopy; SVT, supraventricular tachycardia; VE, ventricular ectopy; VT, ventricular tachycardia. *p* > 0.05 for all (chi-square analysis).

There was no difference between the incidence of arrhythmias or the number of arrhythmias (Table 3, Figure 1) in each participant after any exposure as compared with filtered air (or in the case of the ambient exposures, an exposure in the presence of a highly efficient facemask). Similarly, there was no difference in the incidence or number of arrhythmias in the healthy volunteer and patient subgroups when analyzed independently (see Supplemental Material, Tables S1 and S2). One patient with coronary artery disease (73-year-old male with a past medical history of a previous myocardial infarction and hypertension, with a baseline blood pressure of 172/95 mmHg, who was taking aspirin, benazepril, bisoprolol, and simvastatin) had an asymptomatic episode of nonsustained AF lasting 15 sec while walking in central Beijing during his exposure visit (without a facemask). There were no other episodes of AF or flutter identified during the > 12,500 hr of continuous ECGs.

**Table 3:** Occurrence and number of arrhythmias in all participants (*n* = 282) included in the study (OR of arrhythmia occurring after pollutant exposure as compared with unexposed air controls).

Exposure; arrhythmia	No. of participants with documented arrhythmia		OR (95% CI)	*p*-Value	No. of events per participant [median (IQR)]		*p*-Value
	Unexposed	Exposed			Unexposed	Exposed	
Diesel exhaust (*n *= 117)
Pause	2	1	0.50 (0.04–5.55)	0.56	0 (0–0)	0 (0–0)	> 0.99
Dropped beat	36	37	1.04 (0.60–1.81)	0.89	0 (0–1)	0 (0–1)	0.72
VT	1	0	0.33 (0.01–8.20)	0.32	0 (0–0)	0 (0–0)	> 0.99
Salvo	1	4	4.11 (0.45–37.32)	0.18	0 (0–0)	0 (0–0)	0.56
Triplet	1	3	3.05 (0.31–29.80)	0.31	0 (0–0)	0 (0–0)	> 0.99
Couplet	9	11	1.25 (0.50–3.13)	0.64	0 (0–0)	0 (0–0)	> 0.99
Bradycardia	60	57	0.90 (0.54–1.51)	0.69	1 (0–57)	0 (0–35.5)	0.30
SVT	2	2	1.00 (0.14–7.22)	1.00	0 (0–0)	0 (0–0)	> 0.99
AF	0	0	NA	NA	NA	NA	NA
Bigeminy	7	3	0.41 (0.10–1.64)	0.20	0 (0–0)	0 (0–0)	0.96
Trigeminy	4	4	1.00 (0.24–4.10)	1.00	0 (0–0)	0 (0–0)	0.50
VE	58	54	0.87 (0.52–1.46)	0.60	1 (0–4)	0 (0–4.5)	0.75
SVE	61	65	1.15 (0.69–1.92)	0.60	1 (0–6)	0 (0–4.5)	0.90
Ambient (*n *= 107)
Pause	0	0	NA	NA	NA	NA	NA
Dropped beat	1	2	2.02 (0.18–22.62)	0.56	0 (0–0)	0 (0–0)	> 0.99
VT	1	2	2.02 (0.18–22.62)	0.56	0 (0–0)	0 (0–0)	> 0.99
Salvo	1	2	2.02 (0.18–22.62)	0.56	0 (0–0)	0 (0–0)	> 0.99
Triplet	1	0	0.33 (0.01–8.20)	0.32	0 (0–0)	0 (0–0)	> 0.99
Couplet	9	4	0.42 (0.13–1.42)	0.15	0 (0–0)	0 (0–0)	0.24
Bradycardia	25	21	0.8 (0.42–1.54)	0.51	0 (0–0)	0 (0–0)	0.82
SVT	2	5	2.57 (0.49–13.57)	0.25	0 (0–0)	0 (0–0)	0.45
AF	0	1	3.03 (0.12–75.34)	0.32	0 (0–0)	0 (0–0)	> 0.99
Bigeminy	16	18	1.15 (0.55–2.40)	0.71	0 (0–0)	0 (0–0)	0.80
Trigeminy	4	5	1.26 (0.33–4.84)	0.73	0 (0–0)	0 (0–0)	0.50
VE	87	86	0.94 (0.48–1.86)	0.86	7 (1–66)	7 (1–83)	0.52
SVE	86	88	1.13 (0.57–2.25)	0.73	5 (1–28)	6 (1–28)	0.25
CAPs (*n *= 29)	
Pause	1	0	0.32 (0.01–8.24)	0.31	0 (0–0)	0 (0–0)	> 0.99
Dropped beat	5	5	1.00 (0.26–3.91)	1.00	0 (0–0)	0 (0–0)	0.73
VT	0	0	NA	NA	NA	NA	NA
Salvo	1	0	0.32 (0.01–8.24)	0.31	0 (0–0)	0 (0–0)	> 0.99
Triplet	2	4	2.16 (0.36–12.85)	0.39	0 (0–0)	0 (0–0)	> 0.99
Couplet	2	3	1.56 (0.24–10.10	0.64	0 (0–0)	0 (0–0)	> 0.99
Bradycardia	16	14	0.76 (0.27–2.13)	0.60	2 (0–33)	0 (0–28.5)	0.42
SVT	0	0	NA	NA	NA	NA	NA
AF	0	0	NA	NA	NA	NA	NA
Bigeminy	2	1	0.48 (0.04–5.64)	0.55	0 (0–0)	0 (0–0)	> 0.99
Trigeminy	1	2	2.07 (0.18–24.24)	0.55	0 (0–0)	0 (0–0)	> 0.99
VE	22	26	2.76 (0.64–11.96)	0.16	5 (0.5–34.5)	4 (2–28.5)	0.93
SVE	23	23	1.00 (0.28–3.56)	1.00	2 (1–8)	4 (1–13)	0.06
Wood smoke (*n *= 29)
Pause	2	0	0.19 (0.01–4.06)	0.15	0 (0–0)	0 (0–0)	0.50
Dropped beat	7	9	1.41 (0.44–4.51)	0.56	0 (0–0.5)	0 (0–1)	0.17
VT	0	0	NA	NA	NA	NA	NA
Salvo	0	0	NA	NA	NA	NA	NA
Triplet	0	0	NA	NA	NA	NA	NA
Couplet	0	0	NA	NA	NA	NA	NA
Bradycardia	18	18	1.00 (0.35–2.90)	1.00	3 (0–113)	5 (0–87.5)	0.32
SVT	0	0	NA	NA	NA	NA	NA
AF	0	0	NA	NA	NA	NA	NA
Bigeminy	1	0	0.32 (0.01–8.24)	0.31	0 (0–0)	0 (0–0)	> 0.99
Trigeminy	1	0	0.32 (0.01–8.24)	0.31	0 (0–0)	0 (0–0)	> 0.99
VE	19	15	0.56 (0.20–1.62)	0.29	0 (0–1)	0 (0–1)	0.07
SVE	17	14	0.66 (0.23–1.86)	0.43	3 (1–10.5)	2 (0–7.5)	0.79
Engineered carbon NPs (*n *= 14)	
Pause	0	0	NA	NA	NA	NA	NA
Dropped beat	14	12	0.17 (0.01–3.94)	0.14	6 (2.75–14)	6.5 (3.25–13.75)	0.88
VT	0	0	NA	NA	NA	NA	NA
Salvo	0	0	NA	NA	NA	NA	NA
Triplet	0	0	NA	NA	NA	NA	NA
Couplet	0	0	NA	NA	NA	NA	NA
Bradycardia	7	6	0.75 (0.17–3.32)	0.70	0.5 (0–64)	0 (0–26)	0.74
SVT	0	0	NA	NA	NA	NA	NA
AF	0	0	NA	NA	NA	NA	NA
Bigeminy	0	0	NA	NA	NA	NA	NA
Trigeminy	0	0	NA	NA	NA	NA	NA
VE	7	9	1.80 (0.40–8.19)	0.45	0.5 (0–7.25)	1 (0–5.5)	0.64
SVE	9	10	1.39 (0.28–6.84)	0.69	2.5 (0–5)	2 (0–5)	0.42
O_3_^–^ (*n *= 15)
Pause	1	1	1.00 (0.06–17.63)	1.00	0 (0–0)	0 (0–0)	> 0.99
Dropped beat	5	8	2.29 (0.52–10.01)	0.27	0 (0–1)	1 (0–4)	0.23
VT	0	0	NA	NA	NA	NA	NA
Salvo	0	0	NA	NA	NA	NA	NA
Triplet	0	0	NA	NA	NA	NA	NA
Couplet	0	0	NA	NA	NA	NA	NA
Bradycardia	12	11	0.69 (0.12–3.79)	0.67	18 (1–271)	14 (0–33)	0.12
SVT	0	0	NA	NA	NA	NA	NA
AF	0	0	NA	NA	NA	NA	NA
Bigeminy	0	0	NA	NA	NA	NA	NA
Trigeminy	0	0	NA	NA	NA	NA	NA
VE	12	8	0.29 (0.06–1.44)	0.25	2 (1–3)	1 (0–2)	0.21
SVE	9	12	2.67 (0.52–13.66)	0.23	1 (0–3)	1 (1–3)	0.86

## Discussion

We have compiled the single largest series of studies documenting continuous ECG monitoring in healthy volunteers and patients with stable coronary heart disease on appropriate medical therapy, who have been exposed to a diverse range of environmental air pollutants in acute controlled-exposure studies. In > 12,500 hr of ECG data, we identified no evidence to suggest an increased tendency to arrhythmia after brief controlled exposures. These data indicate that there is no significant risk of arrhythmia associated with controlled exposure to a wide range of air pollutants.

*Air pollution and risk of arrhythmia*. The epidemiological data linking exposure to air pollution and arrhythmia is limited. In a recent study conducted in Taipei, Taiwan, the total hospital admissions with cardiac arrhythmia over a 4-year period (> 16,000 hospital visits) were associated with daily increases in PM air pollution (Chiu et al. 2013), although the investigators provided no information on the type of arrhythmias observed. Among patients with implantable cardiac defibrillators, some studies have reported an increase in ventricular arrhythmias with increasing exposure to particulate air pollutants (Dockery et al. 2005; Ljungman et al. 2008; Peters et al. 2000; Rich et al. 2006). In a recent study of elderly patients with coronary heart disease, in which 20% of participants had a history of congestive cardiac failure, there was a small increase in the risk of nonsustained ventricular tachycardia measured on ambulatory ECG with increasing exposure to PM air pollution (Bartell et al. 2013), although the same study did not find associations with changes in HRV or supraventricular arrhythmias. The finding of an increased risk of ventricular arrhythmia is, however, not consistent, and others have failed to show similar associations (Anderson et al. 2010; Metzger et al. 2007; Rich et al. 2004; Vedal et al. 2004). In a recent long-term follow-up from the Normative Aging Study, short-term exposure to combustion-derived PM air pollution (measured as black carbon) was associated with an increased risk of ventricular ectopy (Zanobetti et al. 2014). There is an association between air pollution exposure and the risk of hospitalization due to cardiac dysrhythmia (Colais et al. 2012; Santos et al. 2008; Tsai et al. 2009) and out-of-hospital cardiac arrest (Rosenthal et al. 2013), although this may be confounded by the strong association between exposure and the triggering of myocardial infarction (Nawrot et al. 2011) or decompensation of patients with cardiac failure (Atkinson et al. 2013; Shah et al. 2013).

Although air pollution exposure is robustly linked to changes in cardiac autonomic nervous system activity (Pieters et al. 2012), which in turn may alter atrial electrical properties and increase the risk of atrial arrhythmia (Arora 2012; Lo et al. 2011; Park et al. 2012), the association between air pollutant exposure and supraventricular arrhythmia is less robust. Among patients with coronary heart disease and elderly participants, increasing exposure to PM air pollution increased the incidence of asymptomatic runs of supraventricular arrhythmias in two observational studies (Berger et al. 2006; Sarnat et al. 2006). However, in a recent robust case-crossover study of > 10,000 admissions to hospital with AF, there was no association with PM air pollution (Bunch et al. 2011). A more recent prospective analysis of patients with implantable cardiac defibrillators with established cardiac disease showed an increased risk of AF with acute increases in exposure to PM air pollution (Link et al. 2013). These contrasting findings may reflect the underlying individual susceptibility to arrhythmia of the patients recruited into the trials. Positive associations have generally been in patients with established cardiac disease, most notably cardiac failure who have structural abnormalities of the cardiac muscle and are generally at increased risk of developing cardiac dysrhythmias. Indeed, we have recently observed an increased risk of hospitalization and death with increasing PM air pollution exposure in patients with heart failure (Shah et al. 2013).

We previously reported that among 32 healthy volunteers and 20 patients exposed to dilute diesel exhaust in controlled-exposure studies, there were no increases in cardiac arrhythmia or changes in HRV (Mills et al. 2011a), and the findings from the present study are similar. Our screening procedures ensured all healthy volunteers were free from cardiac disease, and we excluded patients with coronary heart disease who had resting ECG abnormalities or a history of arrhythmia. Therefore, we studied a relatively low-risk population. We cannot exclude an effect of exposure to air pollutants in patients with overt cardiac failure who have conditional susceptibility to developing arrhythmias.

In their recent case report, Ghio et al. (2012) described a 58-year-old hypertensive female volunteer with frequent atrial ectopy who developed sustained AF/flutter during exposure to CAPs. The authors suggested a causal link; however, AF is the most common cardiac arrhythmia in the general population and is associated with increasing age, hypertension, and cardiac dysfunction and may be triggered by atrial ectopic beats originating from within the pulmonary veins (Haissaguerre et al. 1998). We suggest that it is more likely that the investigators simply witnessed an asymptomatic episode of AF in a patient at increased arrhythmic risk due to coexistent hypertension, age, and frequent atrial ectopy, and the occurrence of AF in the exposure chamber is likely to have been coincidence and simply due to chance. The short (< 0.25 min) single episode of asymptomatic AF in > 750,000 min of ECG recordings in our studies is similarly likely to be due to chance in a patient at risk of arrhythmia due to poorly controlled hypertension and ischaemic heart disease.

*Controlled-exposure studies*. Air pollution research is challenging due to the ever-changing ambient concentrations and composition of the air pollution mixture. Controlled-exposure studies in both animal models and humans have been employed to address fundamental questions necessary to understand the association between air pollution exposure and acute cardiorespiratory effects (Langrish et al. 2010). These studies remain crucial when it comes to identifying important pathophysiological pathways involved in the adverse effects of air pollution. Although there is some limited evidence of an association between exposure to PM air pollution and the risk of arrhythmia, particularly in at-risk populations, the individual risk during a short controlled-exposure study is likely to be extremely small. We have shown that such studies do not increase the short-term risk of arrhythmia in healthy volunteers and patient groups thought to have an increased susceptibility to the adverse effects of air pollution, such as those with chronic respiratory conditions and coronary heart disease.

## Conclusions

Our data suggest that acute controlled exposures to air pollutants are safe and do not significantly increase the short-term risk of arrhythmia among individuals at low risk of arrhythmia. Research employing these techniques, when scientifically and ethically justified (National Research Council 2004; Rom et al. 2013), should continue and remains crucial in identifying pathophysiological pathways involved in the adverse effects of air pollution identified at the population level. These studies can be performed with minimal risk and have the potential for substantial societal benefit, informing environmental and public health policy decisions.

## Supplemental Material

(222 KB) PDF
