# Supplementary material for: Controlled Exposures to Air Pollutants and Risk of Cardiac Arrhythmia
Source: Environ Health Perspect. 2014 Mar 25;122(7):747–53. doi: 10.1289/ehp.1307337 (PMC4080532; doi:10.1289/ehp.1307337)
Supplement: (222 KB) PDF [file ehp.1307337.s001.pdf]

Environmental Health Perspectives doi:10.1289/ehp.1307337

Supplemental Material

Controlled Exposures to Air Pollutants and Risk of Cardiac Arrhythmia

Jeremy P. Langrish, Simon J. Watts, Amanda J. Hunter, Anoop S.V. Shah, Jenny A. Bosson, Jon Unosson, Stefan Barath, Magnus Lundbäck, Flemming R. Cassee, Ken Donaldson, Thomas Sandström, Anders Blomberg, David E. Newby, and Nicholas L. Mills

Table S1. Incidence and number of arrhythmias occurring in healthy volunteers during and after exposure to air pollutants. Odds ratio of arrhythmia occurring following pollutant "exposure" as compared to non-exposed "air" controls.

Exposure and arrhythmia	Unexposed: No. subjects with documented arrhythmia	Exposure: No. subjects with documented arrhythmia	Odds Ratio (95% CI)	P Value	Unexposed: No. events per subject [median (IQR)]	Exposure: No. events per subject [median (IQR)]	P value
**Diesel Exhaust (n=80)**							
Pause	2	0	0.20 (0.01-4.13)	0.15	0 (0-0)	0 (0-0)	0.50
Dropped Beat	33	30	0.85 (0.45-1.61)	0.63	0 (0-3)	0 (0-2.75)	0.42
VT	0	0	N/A	N/A	N/A	N/A	N/A
Salvo	0	1	3.04 (0.12-75.75)	0.32	0 (0-0)	0 (0-0)	>0.99
Triplet	0	0	N/A	N/A	N/A	N/A	N/A
Couplet	1	0	0.33 (0.01-8.21)	0.32	0 (0-0)	0 (0-0)	>0.99
Bradycardia	43	43	1.00 (0.54-1.86)	1.00	1 (0-74)	2 (0-66.25)	0.47
SVT	0	0	N/A	N/A	N/A	N/A	N/A
Bigeminy	2	0	0.20 (0.01-4.13)	0.15	0 (0-0)	0 (0-0)	0.50
Trigeminy	0	0	N/A	N/A	N/A	N/A	N/A
VE	37	34	0.86 (0.46-1.60)	0.63	0 (0-2)	0 (0-2)	0.76
SVE	42	46	1.22 (0.66-2.28)	0.53	1 (0-4)	1 (0-3)	0.71
**Ambient (n=14)**			N/A	N/A	N/A	N/A	N/A
Pause	0	0	N/A	N/A	N/A	N/A	N/A
Dropped Beat	0	0	N/A	N/A	N/A	N/A	N/A
VT	0	0	N/A	N/A	N/A	N/A	N/A
Salvo	0	0	N/A	N/A	N/A	N/A	N/A
Triplet	0	0	N/A	N/A	N/A	N/A	N/A
Couplet	0	0	N/A	N/A	N/A	N/A	N/A
Bradycardia	5	2	0.30 (0.05-1.92)	0.19	0 (0-22)	0 (0-0)	0.06
SVT	0	0	N/A	N/A	N/A	N/A	N/A

**Exposure and arrhythmia**	**Unexposed: No. subjects with documented arrhythmia**	**Exposure: No. subjects with documented arrhythmia**	**Odds Ratio (95% CI)**	**P Value**	**Unexposed: No. events per subject [median (IQR)]**	**Exposure: No. events per subject [median (IQR)]**	**P value**
Bigeminy	1	1	1.00 (0.06-17.76)	1.00	0 (0-0)	0 (0-0)	>0.99
Trigeminy	1	1	1.00 (0.06-17.76)	1.00	0 (0-0)	0 (0-0)	>0.99
VE	5	6	1.35 (0.30-6.19)	0.70	0 (0-2)	0 (0-1.25)	0.46
SVE	7	6	0.75 (0.17-3.33)	0.70	0.5 (0-1.25)	0 (0-1)	0.43
**Concentrated Ambient Particles (n=17)**							
Pause	1	0	0.31 (0.01-8.28)	0.31	0 (0-0)	0 (0-0)	>0.99
Dropped Beat	2	4	2.31 (0.36-14.72)	0.37	0 (0-0)	0 (0-0)	0.63
VT	0	0	N/A	N/A	N/A	N/A	N/A
Salvo	0	0	N/A	N/A	N/A	N/A	N/A
Triplet	0	2	5.65 (0.25-127.00)	0.14	0 (0-0)	0 (0-0)	0.50
Couplet	0	1	3.18 (0.12-83.83)	0.31	0 (0-0)	0 (0-0)	>0.99
Bradycardia	11	8	0.48 (0.12-1.92)	0.30	4 (0-142)	0 (0-62)	0.25
SVT	0	0	N/A	N/A	N/A	N/A	N/A
Bigeminy	1	0	0.31 (0.01-8.28)	0.31	0 (0-0)	0 (0-0)	>0.99
Trigeminy	0	0	N/A	N/A	N/A	N/A	N/A
VE	12	14	1.94 (0.38-9.89)	0.42	4 (0-17.5)	3 (1-7)	0.84
SVE	11	13	1.77 (0.40-7.94)	0.45	1 (0-4)	1 (0.5-7.5)	0.25
**Wood smoke (n=29)**							
Pause	2	0	0.19 (0.01-4.06)	0.15	0 (0-0)	0 (0-0)	0.50
Dropped Beat	7	9	1.41 (0.44-4.51)	0.56	0 (0-0.5)	0 (0-1)	0.17
VT	0	0	N/A	N/A	N/A	N/A	N/A
Salvo	0	0	N/A	N/A	N/A	N/A	N/A
Triplet	0	0	N/A	N/A	N/A	N/A	N/A
Couplet	0	0	N/A	N/A	N/A	N/A	N/A
Bradycardia	18	18	1.00 (0.35-2.90)	1.00	3 (0-113)	5 (0-87.5)	0.32

Exposure and arrhythmia	Unexposed: No. subjects with documented arrhythmia	Exposure: No. subjects with documented arrhythmia	Odds Ratio (95% CI)	P Value	Unexposed: No. events per subject [median (IQR)]	Exposure: No. events per subject [median (IQR)]	P value
SVT	0	0	N/A	N/A	N/A	N/A	N/A
Bigeminy	1	0	0.32 (0.01-8.24)	0.31	0 (0-0)	0 (0-0)	>0.99
Trigeminy	1	0	0.32 (0.01-8.24)	0.31	0 (0-0)	0 (0-0)	>0.99
VE	19	15	0.56 (0.20-1.62)	0.29	0 (0-1)	0 (0-1)	0.07
SVE	17	14	0.66 (0.23-1.86)	0.43	3 (1-10.5)	2 (0-7.5)	0.79
**Engineered carbon nanoparticles (n=14)**							
Pause	0	0	N/A	N/A	N/A	N/A	N/A
Dropped Beat	14	12	0.17 (0.01-3.94)	0.14	6 (2.75-14)	6.5 (3.25-13.75)	0.88
VT	0	0	N/A	N/A	N/A	N/A	N/A
Salvo	0	0	N/A	N/A	N/A	N/A	N/A
Triplet	0	0	N/A	N/A	N/A	N/A	N/A
Couplet	0	0	N/A	N/A	N/A	N/A	N/A
Bradycardia	7	6	0.75 (0.17-3.32)	0.70	0.5 (0-64)	0 (0-26)	0.74
SVT	0	0	N/A	N/A	N/A	N/A	N/A
Bigeminy	0	0	N/A	N/A	N/A	N/A	N/A
Trigeminy	0	0	N/A	N/A	N/A	N/A	N/A
VE	7	9	1.80 (0.40-8.19)	0.45	0.5 (0-7.25)	1 (0-5.5)	0.64
SVE	9	10	1.39 (0.28-6.84)	0.69	2.5 (0-5)	2 (0-5)	0.42
**Ozone (n=15)**							
Pause	1	1	1.00 (0.06-17.63)	1.00	0 (0-0)	0 (0-0)	>0.99
Dropped Beat	5	8	2.29 (0.52-10.01)	0.27	0 (0-1)	1 (0-4)	0.23
VT	0	0	N/A	N/A	N/A	N/A	N/A
Salvo	0	0	N/A	N/A	N/A	N/A	N/A
Triplet	0	0	N/A	N/A	N/A	N/A	N/A
Couplet	0	0	N/A	N/A	N/A	N/A	N/A

Exposure and arrhythmia	Unexposed: No. subjects with documented arrhythmia	Exposure: No. subjects with documented arrhythmia	Odds Ratio (95% CI)	P Value	Unexposed: No. events per subject [median (IQR)]	Exposure: No. events per subject [median (IQR)]	P value
Bradycardia	12	11	0.69 (0.12-3.79)	0.67	18 (1-271)	14 (0-33)	0.12
SVT	0	0	N/A	N/A	N/A	N/A	N/A
Bigeminy	0	0	N/A	N/A	N/A	N/A	N/A
Trigeminy	0	0	N/A	N/A	N/A	N/A	N/A
VE	12	8	0.29 (0.06-1.44)	0.25	2 (1-3)	1 (0-2)	0.21
SVE	9	12	2.67 (0.52-13.66)	0.23	1 (0-3)	1 (1-3)	0.86

VT = ventricular tachycardia; SVT = supraventricular tachycardia; VE = ventricular ectopic beat; SVE = supraventricular ectopic beat; bradycardia defined as HR <50 bpm.

Data expressed as number or median (interquartile range) as appropriate.

P values and odds ratios from Chi-squared analysis and Wilcoxon Matched-Pairs Signed Rank Test as appropriate.

Table S2. Incidence and number of arrhythmias in the patients with coronary heart disease during and after exposure to air pollutants. Odds ratio of arrhythmia occurring following pollutant "exposure" as compared to non-exposed "air" controls.

Exposure and arrhythmia	Unexposed: No. subjects with documented arrhythmia	Exposure: No. subjects with documented arrhythmia	Odds Ratio (95% CI)	P Value	Unexposed: No. events per subject [median (IQR)]	Exposure: No. events per subject [median (IQR)]	P value
**Diesel Exhaust (n=37)**							
Pause	0	1	3.08 (0.12-78.20)	0.31	0 (0-0)	0 (0-0)	>0.99
Dropped Beat	3	7	2.64 (0.63-11.15)	0.17	0 (0-0)	0 (0-0)	0.05
VT	1	0	0.32 (0.01-8.23)	0.31	0 (0-0)	0 (0-0)	>0.99
Salvo	1	3	3.18 (0.31-32.06)	0.30	0 (0-0)	0 (0-0)	0.75
Triplet	1	3	3.18 (0.31-32.06)	0.30	0 (0-0)	0 (0-0)	>0.99
Couplet	8	11	1.53 (0.54-4.40)	0.42	0 (0-0)	0 (0-1)	>0.99
Bradycardia	17	14	0.72 (0.28-1.81)	0.48	0 (0-40.5)	0 (0-5.5)	0.37
SVT	2	2	1.00 (0.13-7.51)	1.00	0 (0-0)	0 (0-0)	>0.99
Bigeminy	5	3	0.56 (0.13-2.56)	0.45	0 (0-0)	0 (0-0)	0.68
Trigeminy	4	4	1.00 (0.23-4.34)	1.00	0 (0-0)	0 (0-0)	0.50
VE	25	24	0.89 (0.34-2.32)	0.81	4 (0-37)	3 (0-30.5)	0.84
SVE	19	19	1.00 (0.40-2.49)	1.00	1 (0-25.5)	1 (0-24)	0.77
**Ambient (n=93)**							
Pause	0	0	N/A	N/A	N/A	N/A	N/A
Dropped Beat	1	2	2.02 (0.18-22.70)	0.56	0 (0-0)	0 (0-0)	>0.99
VT	1	2	2.02 (0.18-22.70)	0.56	0 (0-0)	0 (0-0)	>0.99
Salvo	1	2	2.02 (0.18-22.70)	0.56	0 (0-0)	0 (0-0)	>0.99
Triplet	1	0	0.33 (0.01-8.21)	0.32	0 (0-0)	0 (0-0)	>0.99
Couplet	9	4	0.42 (0.12-1.41)	0.15	0 (0-0)	0 (0-0)	0.14
Bradycardia	20	19	0.94 (0.46-1.90)	0.86	0 (0-0)	0 (0-0)	0.71
SVT	2	5	2.59 (0.49-13.68)	0.25	0 (0-0)	0 (0-0)	0.45
Bigeminy	15	17	1.16 (0.54-2.50)	0.70	0 (0-0)	0 (0-0)	0.54

Exposure and arrhythmia	Unexposed: No. subjects with documented arrhythmia	Exposure: No. subjects with documented arrhythmia	Odds Ratio (95% CI)	P Value	Unexposed: No. events per subject [median (IQR)]	Exposure: No. events per subject [median (IQR)]	P value
Trigeminy	3	4	1.35 (0.29-6.20)	0.70	0 (0-0)	0 (0-0)	0.25
VE	82	80	0.83 (0.35-1.95)	0.66	9 (2-103)	11 (2-95)	0.34
SVE	79	82	1.32 (0.57-3.09)	0.52	8 (1.5-34.5)	8 (1-38)	0.28
**Concentrated Ambient Particles (n=12)**							
Pause	0	0	N/A	N/A	N/A	N/A	N/A
Dropped Beat	3	1	0.27 (0.02-3.10)	0.27	0 (0-0)	0 (0-0)	>0.99
VT	0	0	N/A	N/A	N/A	N/A	N/A
Salvo	1	0	0.31 (0.01-8.32)	0.31	0 (0-0)	0 (0-0)	>0.99
Triplet	2	2	1.00 (0.12-8.56)	1.00	0 (0-0)	0 (0-0)	>0.99
Couplet	2	2	1.00 (0.12-8.56)	1.00	0 (0-0)	0 (0-0)	>0.99
Bradycardia	5	6	1.4 (0.28-7.02)	0.68	0 (0-33)	2.5 (0-12.5)	0.94
SVT	0	0	N/A	N/A	N/A	N/A	N/A
Bigeminy	1	1	1.00 (0.06-18.10)	1.00	0 (0-0)	0 (0-0)	>0.99
Trigeminy	1	2	2.20 (0.17-28.16)	0.54	0 (0-0)	0 (0-0)	>0.99
VE	10	12	5.95 (0.26-138.40)	0.14	18.5 (2-181.5)	23.25 (3.25-133.3)	0.90
SVE	12	10	0.17 (0.01-3.91)	0.14	4 (2-16.25)	10.5 (1.75-29)	0.16

VT = ventricular tachycardia; SVT = supraventricular tachycardia; VE = ventricular ectopic beat; SVE = supraventricular ectopic beat; bradycardia defined as HR <50 bpm.

Data expressed as number or median (interquartile range) as appropriate.

P values and odds ratios from Chi-squared analysis and Wilcoxon Matched-Pairs Signed Rank Test as appropriate.
